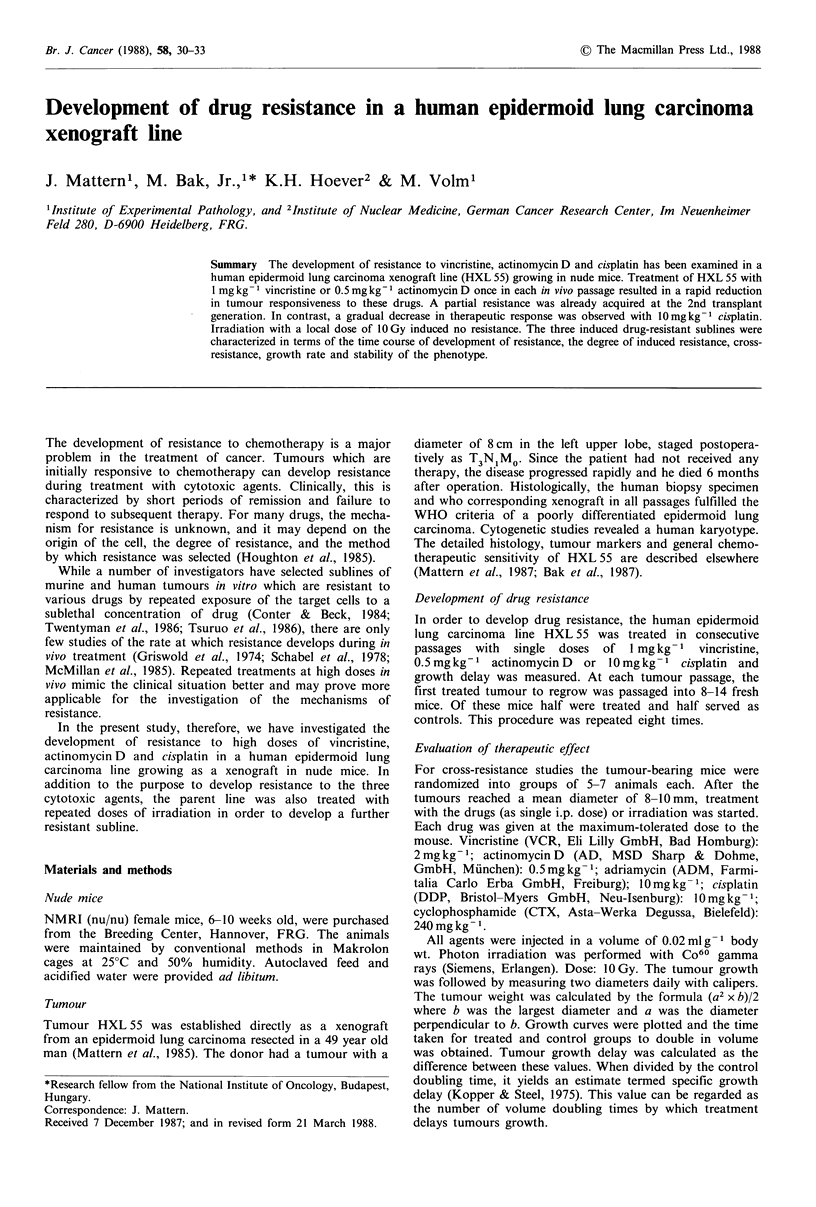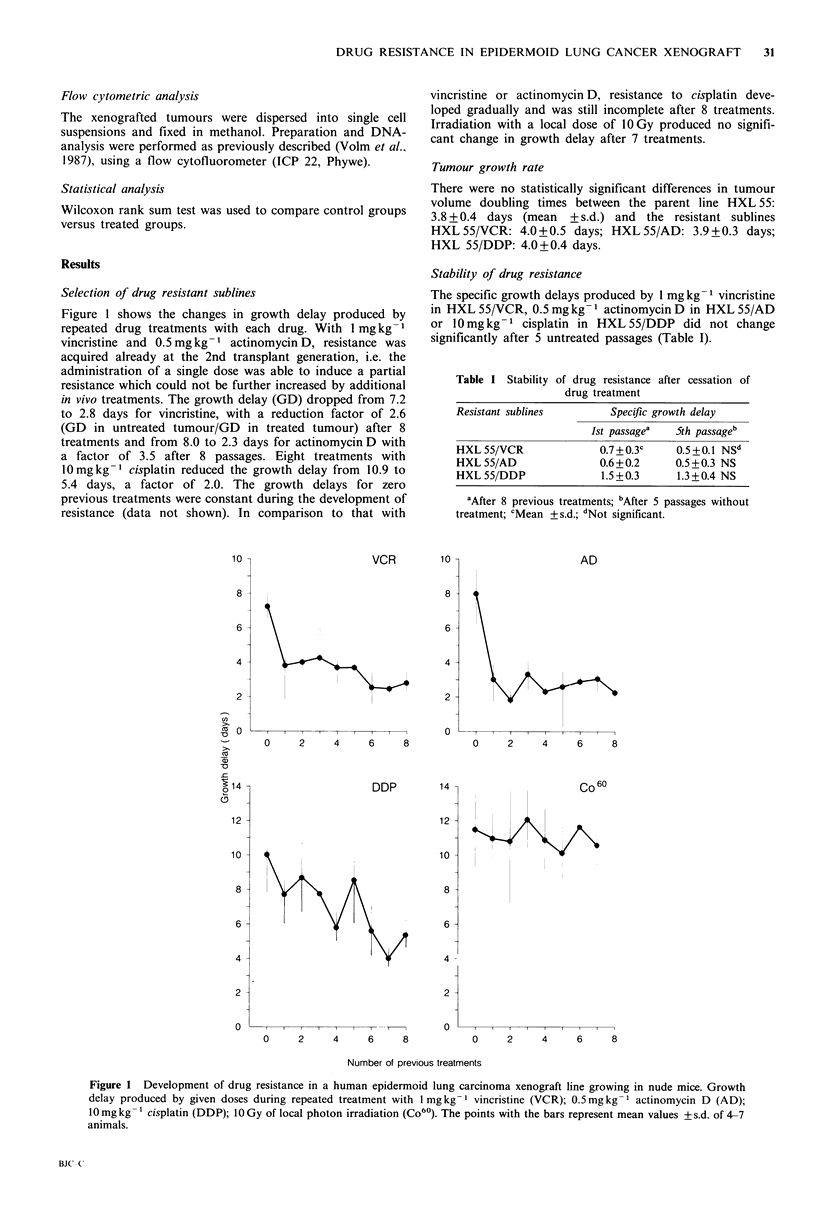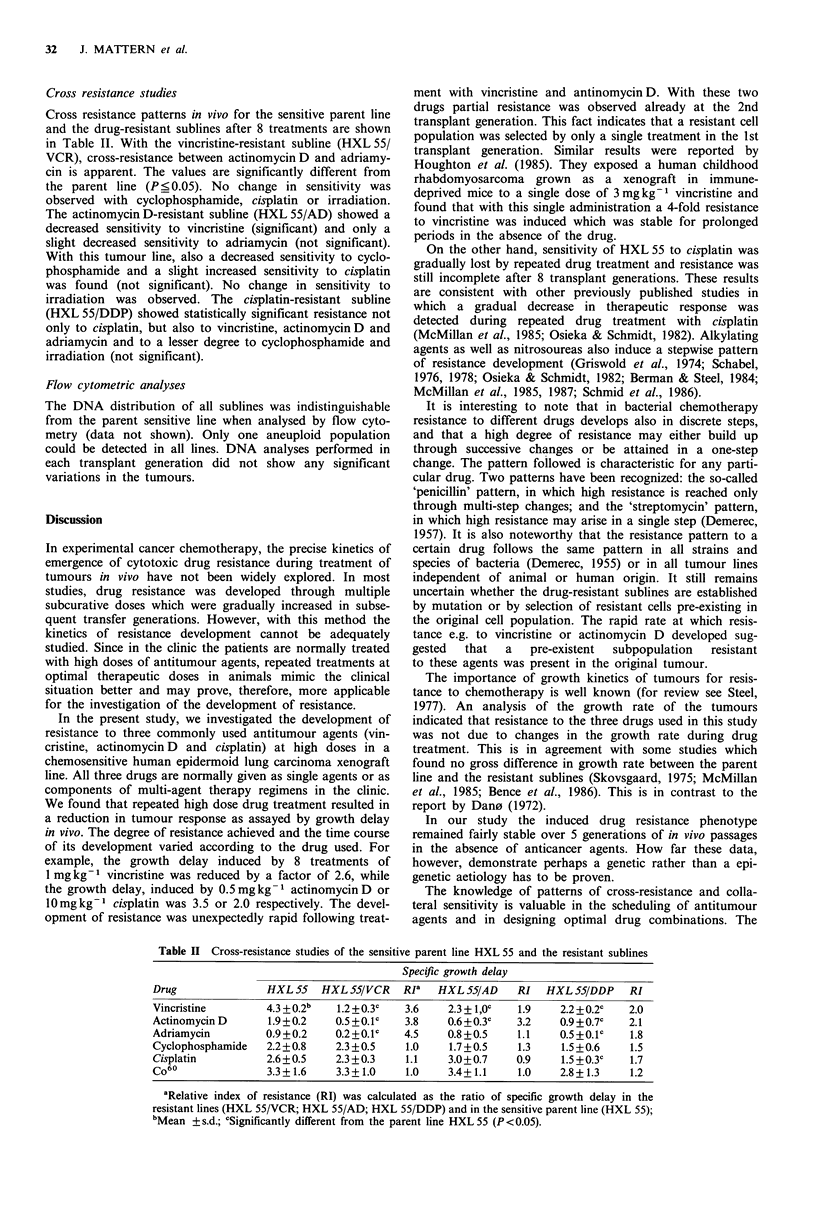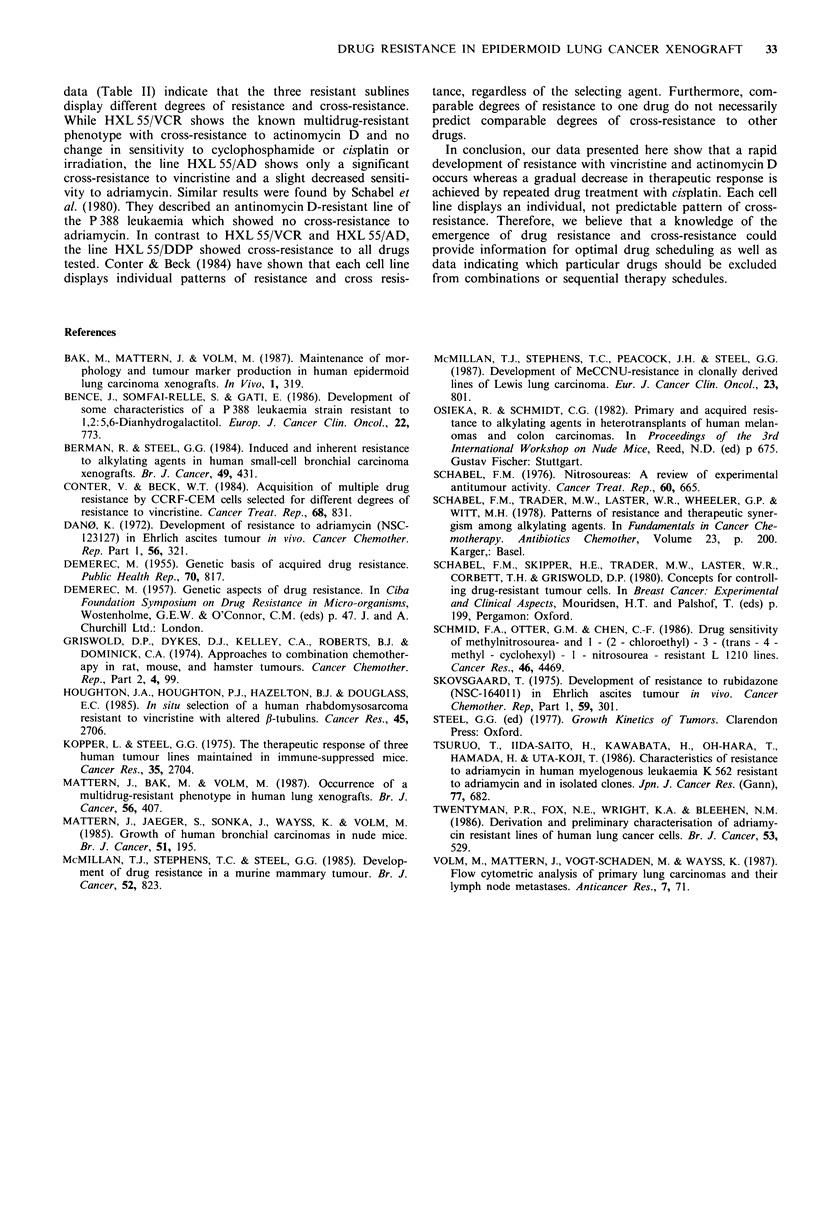# Development of drug resistance in a human epidermoid lung carcinoma xenograft line.

**DOI:** 10.1038/bjc.1988.155

**Published:** 1988-07

**Authors:** J. Mattern, M. Bak, K. H. Hoever, M. Volm

**Affiliations:** Institute of Experimental Pathology, German Cancer Research Center, Heidelberg.

## Abstract

The development of resistance to vincristine, actinomycin D and cisplatin has been examined in a human epidermoid lung carcinoma xenograft line (HXL 55) growing in nude mice. Treatment of HXL 55 with 1 mg kg-1 vincristine or 0.5 mg kg-1 actinomycin D once in each in vivo passage resulted in a rapid reduction in tumour responsiveness to these drugs. A partial resistance was already acquired at the 2nd transplant generation. In contrast, a gradual decrease in therapeutic response was observed with 10 mg kg-1 cisplatin. Irradiation with a local dose of 10 Gy induced no resistance. The three induced drug-resistant sublines were characterized in terms of the time course of development of resistance, the degree of induced resistance, cross-resistance, growth rate and stability of the phenotype.


Br. J. Cancer (1988), 58, 30–33 © The Macmillan Press Ltd., 1988

### Development of drug resistance in a human epidermoid lung carcinoma xenograft line

J. Mattern[1], M. Bak, Jr.,[1]* K.H. Hoever[2] & M. Volm[1]

[1]*Institute of Experimental Pathology, and* [2]*Institute of Nuclear Medicine, German Cancer Research Center, Im Neuenheimer Feld 280, D-6900 Heidelberg, FRG.*

**Summary** The development of resistance to vincristine, actinomycin D and *cis*platin has been examined in a human epidermoid lung carcinoma xenograft line (HXL 55) growing in nude mice. Treatment of HXL 55 with 1 mg kg$^{-1}$ vincristine or 0.5 mg kg$^{-1}$ actinomycin D once in each *in vivo* passage resulted in a rapid reduction in tumour responsiveness to these drugs. A partial resistance was already acquired at the 2nd transplant generation. In contrast, a gradual decrease in therapeutic response was observed with 10 mg kg$^{-1}$ *cis*platin. Irradiation with a local dose of 10 Gy induced no resistance. The three induced drug-resistant sublines were characterized in terms of the time course of development of resistance, the degree of induced resistance, cross-resistance, growth rate and stability of the phenotype.

The development of resistance to chemotherapy is a major problem in the treatment of cancer. Tumours which are initially responsive to chemotherapy can develop resistance during treatment with cytotoxic agents. Clinically, this is characterized by short periods of remission and failure to respond to subsequent therapy. For many drugs, the mechanism for resistance is unknown, and it may depend on the origin of the cell, the degree of resistance, and the method by which resistance was selected (Houghton *et al.*, 1985).

While a number of investigators have selected sublines of murine and human tumours *in vitro* which are resistant to various drugs by repeated exposure of the target cells to a sublethal concentration of drug (Conter & Beck, 1984; Twentyman *et al.*, 1986; Tsuruo *et al.*, 1986), there are only few studies of the rate at which resistance develops during *in vivo* treatment (Griswold *et al.*, 1974; Schabel *et al.*, 1978; McMillan *et al.*, 1985). Repeated treatments at high doses *in vivo* mimic the clinical situation better and may prove more applicable for the investigation of the mechanisms of resistance.

In the present study, therefore, we have investigated the development of resistance to high doses of vincristine, actinomycin D and *cis*platin in a human epidermoid lung carcinoma line growing as a xenograft in nude mice. In addition to the purpose to develop resistance to the three cytotoxic agents, the parent line was also treated with repeated doses of irradiation in order to develop a further resistant subline.

### Materials and methods

### *Nude mice*

NMRI (nu/nu) female mice, 6–10 weeks old, were purchased from the Breeding Center, Hannover, FRG. The animals were maintained by conventional methods in Makrolon cages at 25°C and 50% humidity. Autoclaved feed and acidified water were provided *ad libitum.*

### *Tumour*

Tumour HXL 55 was established directly as a xenograft from an epidermoid lung carcinoma resected in a 49 year old man (Mattern *et al.*, 1985). The donor had a tumour with a diameter of 8 cm in the left upper lobe, staged postoperatively as $T_3N_1M_0$. Since the patient had not received any therapy, the disease progressed rapidly and he died 6 months after operation. Histologically, the human biopsy specimen and who corresponding xenograft in all passages fulfilled the WHO criteria of a poorly differentiated epidermoid lung carcinoma. Cytogenetic studies revealed a human karyotype. The detailed histology, tumour markers and general chemotherapeutic sensitivity of HXL 55 are described elsewhere (Mattern *et al.*, 1987; Bak *et al.*, 1987).

### *Development of drug resistance*

In order to develop drug resistance, the human epidermoid lung carcinoma line HXL 55 was treated in consecutive passages with single doses of 1 mg kg$^{-1}$ vincristine, 0.5 mg kg$^{-1}$ actinomycin D or 10 mg kg$^{-1}$ *cis*platin and growth delay was measured. At each tumour passage, the first treated tumour to regrow was passaged into 8–14 fresh mice. Of these mice half were treated and half served as controls. This procedure was repeated eight times.

### *Evaluation of therapeutic effect*

For cross-resistance studies the tumour-bearing mice were randomized into groups of 5–7 animals each. After the tumours reached a mean diameter of 8–10 mm, treatment with the drugs (as single i.p. dose) or irradiation was started. Each drug was given at the maximum-tolerated dose to the mouse. Vincristine (VCR, Eli Lilly GmbH, Bad Homburg): 2 mg kg$^{-1}$; actinomycin D (AD, MSD Sharp & Dohme, GmbH, München): 0.5 mg kg$^{-1}$; adriamycin (ADM, Farmitalia Carlo Erba GmbH, Freiburg); 10 mg kg$^{-1}$; *cis*platin (DDP, Bristol-Myers GmbH, Neu-Isenburg): 10 mg kg$^{-1}$; cyclophosphamide (CTX, Asta-Werka Degussa, Bielefeld): 240 mg kg$^{-1}$.

All agents were injected in a volume of 0.02 ml g$^{-1}$ body wt. Photon irradiation was performed with $Co^{60}$ gamma rays (Siemens, Erlangen). Dose: 10 Gy. The tumour growth was followed by measuring two diameters daily with calipers. The tumour weight was calculated by the formula $(a^2 \times b)/2$ where $b$ was the largest diameter and $a$ was the diameter perpendicular to $b$. Growth curves were plotted and the time taken for treated and control groups to double in volume was obtained. Tumour growth delay was calculated as the difference between these values. When divided by the control doubling time, it yields an estimate termed specific growth delay (Kopper & Steel, 1975). This value can be regarded as the number of volume doubling times by which treatment delays tumours growth.

*Research fellow from the National Institute of Oncology, Budapest, Hungary.
Correspondence: J. Mattern.
Received 7 December 1987; and in revised form 21 March 1988.

*Flow cytometric analysis*

The xenografted tumours were dispersed into single cell suspensions and fixed in methanol. Preparation and DNA-analysis were performed as previously described (Volm *et al.*, 1987), using a flow cytofluorometer (ICP 22, Phywe).

*Statistical analysis*

Wilcoxon rank sum test was used to compare control groups versus treated groups.

### Results

*Selection of drug resistant sublines*

Figure 1 shows the changes in growth delay produced by repeated drug treatments with each drug. With 1 mg kg$^{-1}$ vincristine and 0.5 mg kg$^{-1}$ actinomycin D, resistance was acquired already at the 2nd transplant generation, i.e. the administration of a single dose was able to induce a partial resistance which could not be further increased by additional *in vivo* treatments. The growth delay (GD) dropped from 7.2 to 2.8 days for vincristine, with a reduction factor of 2.6 (GD in untreated tumour/GD in treated tumour) after 8 treatments and from 8.0 to 2.3 days for actinomycin D with a factor of 3.5 after 8 passages. Eight treatments with 10 mg kg$^{-1}$ *cis*platin reduced the growth delay from 10.9 to 5.4 days, a factor of 2.0. The growth delays for zero previous treatments were constant during the development of resistance (data not shown). In comparison to that with vincristine or actinomycin D, resistance to *cis*platin developed gradually and was still incomplete after 8 treatments. Irradiation with a local dose of 10 Gy produced no significant change in growth delay after 7 treatments.

*Tumour growth rate*

There were no statistically significant differences in tumour volume doubling times between the parent line HXL 55: 3.8±0.4 days (mean ±s.d.) and the resistant sublines HXL 55/VCR: 4.0±0.5 days; HXL 55/AD: 3.9±0.3 days; HXL 55/DDP: 4.0±0.4 days.

*Stability of drug resistance*

The specific growth delays produced by 1 mg kg$^{-1}$ vincristine in HXL 55/VCR, 0.5 mg kg$^{-1}$ actinomycin D in HXL 55/AD or 10 mg kg$^{-1}$ cisplatin in HXL 55/DDP did not change significantly after 5 untreated passages (Table I).

**Table I** Stability of drug resistance after cessation of drug treatment

| *Resistant sublines* | *Specific growth delay* | |
|---|---|---|
| | *1st passage*[a] | *5th passage*[b] |
| HXL 55/VCR | 0.7±0.3[c] | 0.5±0.1 NS[d] |
| HXL 55/AD | 0.6±0.2 | 0.5±0.3 NS |
| HXL 55/DDP | 1.5±0.3 | 1.3±0.4 NS |

[a]After 8 previous treatments; [b]After 5 passages without treatment; [c]Mean ±s.d.; [d]Not significant.

**Figure 1** Development of drug resistance in a human epidermoid lung carcinoma xenograft line growing in nude mice. Growth delay produced by given doses during repeated treatment with 1 mg kg$^{-1}$ vincristine (VCR); 0.5 mg kg$^{-1}$ actinomycin D (AD); 10 mg kg$^{-1}$ *cis*platin (DDP); 10 Gy of local photon irradiation ($Co^{60}$). The points with the bars represent mean values ±s.d. of 4–7 animals.

### Cross resistance studies

Cross resistance patterns *in vivo* for the sensitive parent line and the drug-resistant sublines after 8 treatments are shown in Table II. With the vincristine-resistant subline (HXL 55/VCR), cross-resistance between actinomycin D and adriamycin is apparent. The values are significantly different from the parent line ($P \leq 0.05$). No change in sensitivity was observed with cyclophosphamide, *cis*platin or irradiation. The actinomycin D-resistant subline (HXL 55/AD) showed a decreased sensitivity to vincristine (significant) and only a slight decreased sensitivity to adriamycin (not significant). With this tumour line, also a decreased sensitivity to cyclophosphamide and a slight increased sensitivity to *cis*platin was found (not significant). No change in sensitivity to irradiation was observed. The *cis*platin-resistant subline (HXL 55/DDP) showed statistically significant resistance not only to *cis*platin, but also to vincristine, actinomycin D and adriamycin and to a lesser degree to cyclophosphamide and irradiation (not significant).

### Flow cytometric analyses

The DNA distribution of all sublines was indistinguishable from the parent sensitive line when analysed by flow cytometry (data not shown). Only one aneuploid population could be detected in all lines. DNA analyses performed in each transplant generation did not show any significant variations in the tumours.

### Discussion

In experimental cancer chemotherapy, the precise kinetics of emergence of cytotoxic drug resistance during treatment of tumours *in vivo* have not been widely explored. In most studies, drug resistance was developed through multiple subcurative doses which were gradually increased in subsequent transfer generations. However, with this method the kinetics of resistance development cannot be adequately studied. Since in the clinic the patients are normally treated with high doses of antitumour agents, repeated treatments at optimal therapeutic doses in animals mimic the clinical situation better and may prove, therefore, more applicable for the investigation of the development of resistance.

In the present study, we investigated the development of resistance to three commonly used antitumour agents (vincristine, actinomycin D and *cis*platin) at high doses in a chemosensitive human epidermoid lung carcinoma xenograft line. All three drugs are normally given as single agents or as components of multi-agent therapy regimens in the clinic. We found that repeated high dose drug treatment resulted in a reduction in tumour response as assayed by growth delay *in vivo*. The degree of resistance achieved and the time course of its development varied according to the drug used. For example, the growth delay induced by 8 treatments of $1\,mg\,kg^{-1}$ vincristine was reduced by a factor of 2.6, while the growth delay, induced by $0.5\,mg\,kg^{-1}$ actinomycin D or $10\,mg\,kg^{-1}$ *cis*platin was 3.5 or 2.0 respectively. The development of resistance was unexpectedly rapid following treatment with vincristine and antinomycin D. With these two drugs partial resistance was observed already at the 2nd transplant generation. This fact indicates that a resistant cell population was selected by only a single treatment in the 1st transplant generation. Similar results were reported by Houghton *et al.* (1985). They exposed a human childhood rhabdomyosarcoma grown as a xenograft in immune-deprived mice to a single dose of $3\,mg\,kg^{-1}$ vincristine and found that with this single administration a 4-fold resistance to vincristine was induced which was stable for prolonged periods in the absence of the drug.

On the other hand, sensitivity of HXL 55 to *cis*platin was gradually lost by repeated drug treatment and resistance was still incomplete after 8 transplant generations. These results are consistent with other previously published studies in which a gradual decrease in therapeutic response was detected during repeated drug treatment with *cis*platin (McMillan *et al.*, 1985; Osieka & Schmidt, 1982). Alkylating agents as well as nitrosoureas also induce a stepwise pattern of resistance development (Griswold *et al.*, 1974; Schabel, 1976, 1978; Osieka & Schmidt, 1982; Berman & Steel, 1984; McMillan *et al.*, 1985, 1987; Schmid *et al.*, 1986).

It is interesting to note that in bacterial chemotherapy resistance to different drugs develops also in discrete steps, and that a high degree of resistance may either build up through successive changes or be attained in a one-step change. The pattern followed is characteristic for any particular drug. Two patterns have been recognized: the so-called 'penicillin' pattern, in which high resistance is reached only through multi-step changes; and the 'streptomycin' pattern, in which high resistance may arise in a single step (Demerec, 1957). It is also noteworthy that the resistance pattern to a certain drug follows the same pattern in all strains and species of bacteria (Demerec, 1955) or in all tumour lines independent of animal or human origin. It still remains uncertain whether the drug-resistant sublines are established by mutation or by selection of resistant cells pre-existing in the original cell population. The rapid rate at which resistance e.g. to vincristine or actinomycin D developed suggested that a pre-existent subpopulation resistant to these agents was present in the original tumour.

The importance of growth kinetics of tumours for resistance to chemotherapy is well known (for review see Steel, 1977). An analysis of the growth rate of the tumours indicated that resistance to the three drugs used in this study was not due to changes in the growth rate during drug treatment. This is in agreement with some studies which found no gross difference in growth rate between the parent line and the resistant sublines (Skovsgaard, 1975; McMillan *et al.*, 1985; Bence *et al.*, 1986). This is in contrast to the report by Danø (1972).

In our study the induced drug resistance phenotype remained fairly stable over 5 generations of *in vivo* passages in the absence of anticancer agents. How far these data, however, demonstrate perhaps a genetic rather than a epigenetic aetiology has to be proven.

The knowledge of patterns of cross-resistance and collateral sensitivity is valuable in the scheduling of antitumour agents and in designing optimal drug combinations. The

**Table II** Cross-resistance studies of the sensitive parent line HXL 55 and the resistant sublines

| | Specific growth delay | | | | | | |
|---|---|---|---|---|---|---|---|
| *Drug* | *HXL 55* | *HXL 55/VCR* | *RI*[a] | *HXL 55/AD* | *RI* | *HXL 55/DDP* | *RI* |
| Vincristine | 4.3±0.2[b] | 1.2±0.3[c] | 3.6 | 2.3±1,0[c] | 1.9 | 2.2±0.2[c] | 2.0 |
| Actinomycin D | 1.9±0.2 | 0.5±0.1[c] | 3.8 | 0.6±0.3[c] | 3.2 | 0.9±0.7[c] | 2.1 |
| Adriamycin | 0.9±0.2 | 0.2±0.1[c] | 4.5 | 0.8±0.5 | 1.1 | 0.5±0.1[c] | 1.8 |
| Cyclophosphamide | 2.2±0.8 | 2.3±0.5 | 1.0 | 1.7±0.5 | 1.3 | 1.5±0.6 | 1.5 |
| *Cis*platin | 2.6±0.5 | 2.3±0.3 | 1.1 | 3.0±0.7 | 0.9 | 1.5±0.3[c] | 1.7 |
| $Co^{60}$ | 3.3±1.6 | 3.3±1.0 | 1.0 | 3.4±1.1 | 1.0 | 2.8±1.3 | 1.2 |

[a]Relative index of resistance (RI) was calculated as the ratio of specific growth delay in the resistant lines (HXL 55/VCR; HXL 55/AD; HXL 55/DDP) and in the sensitive parent line (HXL 55); [b]Mean ±s.d.; [c]Significantly different from the parent line HXL 55 ($P < 0.05$).

data (Table II) indicate that the three resistant sublines display different degrees of resistance and cross-resistance. While HXL 55/VCR shows the known multidrug-resistant phenotype with cross-resistance to actinomycin D and no change in sensitivity to cyclophosphamide or *cis*platin or irradiation, the line HXL 55/AD shows only a significant cross-resistance to vincristine and a slight decreased sensitivity to adriamycin. Similar results were found by Schabel *et al.* (1980). They described an antinomycin D-resistant line of the P 388 leukaemia which showed no cross-resistance to adriamycin. In contrast to HXL 55/VCR and HXL 55/AD, the line HXL 55/DDP showed cross-resistance to all drugs tested. Conter & Beck (1984) have shown that each cell line displays individual patterns of resistance and cross resistance, regardless of the selecting agent. Furthermore, comparable degrees of resistance to one drug do not necessarily predict comparable degrees of cross-resistance to other drugs.

In conclusion, our data presented here show that a rapid development of resistance with vincristine and actinomycin D occurs whereas a gradual decrease in therapeutic response is achieved by repeated drug treatment with *cis*platin. Each cell line displays an individual, not predictable pattern of cross-resistance. Therefore, we believe that a knowledge of the emergence of drug resistance and cross-resistance could provide information for optimal drug scheduling as well as data indicating which particular drugs should be excluded from combinations or sequential therapy schedules.

### References

BAK, M., MATTERN, J. & VOLM, M. (1987). Maintenance of morphology and tumour marker production in human epidermoid lung carcinoma xenografts. *In Vivo*, **1**, 319.

BENCE, J., SOMFAI-RELLE, S. & GATI, E. (1986). Development of some characteristics of a P 388 leukaemia strain resistant to 1,2:5,6-Dianhydrogalactitol. *Europ. J. Cancer Clin. Oncol.*, **22**, 773.

BERMAN, R. & STEEL, G.G. (1984). Induced and inherent resistance to alkylating agents in human small-cell bronchial carcinoma xenografts. *Br. J. Cancer*, **49**, 431.

CONTER, V. & BECK, W.T. (1984). Acquisition of multiple drug resistance by CCRF-CEM cells selected for different degrees of resistance to vincristine. *Cancer Treat. Rep.*, **68**, 831.

DANØ, K. (1972). Development of resistance to adriamycin (NSC-123127) in Ehrlich ascites tumour *in vivo*. *Cancer Chemother. Rep.* Part 1, **56**, 321.

DEMEREC, M. (1955). Genetic basis of acquired drug resistance. *Public Health Rep.*, **70**, 817.

DEMEREC, M. (1957). Genetic aspects of drug resistance. In *Ciba Foundation Symposium on Drug Resistance in Micro-organisms*, Wostenholme, G.E.W. & O'Connor, C.M. (eds) p. 47. J. and A. Churchill Ltd.: London.

GRISWOLD, D.P., DYKES, D.J., KELLEY, C.A., ROBERTS, B.J. & DOMINICK, C.A. (1974). Approaches to combination chemotherapy in rat, mouse, and hamster tumours. *Cancer Chemother. Rep.*, Part 2, **4**, 99.

HOUGHTON, J.A., HOUGHTON, P.J., HAZELTON, B.J. & DOUGLASS, E.C. (1985). *In situ* selection of a human rhabdomysosarcoma resistant to vincristine with altered $\beta$-tubulins. *Cancer Res.*, **45**, 2706.

KOPPER, L. & STEEL, G.G. (1975). The therapeutic response of three human tumour lines maintained in immune-suppressed mice. *Cancer Res.*, **35**, 2704.

MATTERN, J., BAK, M. & VOLM, M. (1987). Occurrence of a multidrug-resistant phenotype in human lung xenografts. *Br. J. Cancer*, **56**, 407.

MATTERN, J., JAEGER, S., SONKA, J., WAYSS, K. & VOLM, M. (1985). Growth of human bronchial carcinomas in nude mice. *Br. J. Cancer*, **51**, 195.

McMILLAN, T.J., STEPHENS, T.C. & STEEL, G.G. (1985). Development of drug resistance in a murine mammary tumour. *Br. J. Cancer*, **52**, 823.

McMILLAN, T.J., STEPHENS, T.C., PEACOCK, J.H. & STEEL, G.G. (1987). Development of MeCCNU-resistance in clonally derived lines of Lewis lung carcinoma. *Eur. J. Cancer Clin. Oncol.*, **23**, 801.

OSIEKA, R. & SCHMIDT, C.G. (1982). Primary and acquired resistance to alkylating agents in heterotransplants of human melanomas and colon carcinomas. In *Proceedings of the 3rd International Workshop on Nude Mice*, Reed, N.D. (ed) p 675. Gustav Fischer: Stuttgart.

SCHABEL, F.M. (1976). Nitrosoureas: A review of experimental antitumour activity. *Cancer Treat. Rep.*, **60**, 665.

SCHABEL, F.M., TRADER, M.W., LASTER, W.R., WHEELER, G.P. & WITT, M.H. (1978). Patterns of resistance and therapeutic synergism among alkylating agents. In *Fundamentals in Cancer Chemotherapy. Antibiotics Chemother*, Volume 23, p. 200. Karger,: Basel.

SCHABEL, F.M., SKIPPER, H.E., TRADER, M.W., LASTER, W.R., CORBETT, T.H. & GRISWOLD, D.P. (1980). Concepts for controlling drug-resistant tumour cells. In *Breast Cancer: Experimental and Clinical Aspects*, Mouridsen, H.T. and Palshof, T. (eds) p. 199, Pergamon: Oxford.

SCHMID, F.A., OTTER, G.M. & CHEN, C.-F. (1986). Drug sensitivity of methylnitrosourea- and 1 - (2 - chloroethyl) - 3 - (trans - 4 - methyl - cyclohexyl) - 1 - nitrosourea - resistant L 1210 lines. *Cancer Res.*, **46**, 4469.

SKOVSGAARD, T. (1975). Development of resistance to rubidazone (NSC-164011) in Ehrlich ascites tumour *in vivo*. *Cancer Chemother. Rep*, Part 1, **59**, 301.

STEEL, G.G. (ed) (1977). *Growth Kinetics of Tumors*. Clarendon Press: Oxford.

TSURUO, T., IIDA-SAITO, H., KAWABATA, H., OH-HARA, T., HAMADA, H. & UTA-KOJI, T. (1986). Characteristics of resistance to adriamycin in human myelogenous leukaemia K 562 resistant to adriamycin and in isolated clones. *Jpn. J. Cancer Res.* (Gann), **77**, 682.

TWENTYMAN, P.R., FOX, N.E., WRIGHT, K.A. & BLEEHEN, N.M. (1986). Derivation and preliminary characterisation of adriamycin resistant lines of human lung cancer cells. *Br. J. Cancer*, **53**, 529.

VOLM, M., MATTERN, J., VOGT-SCHADEN, M. & WAYSS, K. (1987). Flow cytometric analysis of primary lung carcinomas and their lymph node metastases. *Anticancer Res.*, **7**, 71.